# Dual emission from nanoconfined R-phycoerythrin fluorescent proteins for white light emission diodes†

**DOI:** 10.1039/c9ra00161a

**Published:** 2019-03-27

**Authors:** Xiaobin Wang, Yi Guo, Zhuoyi Li, Wen Ying, Danke Chen, Zheng Deng, Xinsheng Peng

**Affiliations:** State Key Laboratory of Silicon Materials, School of Materials Science and Engineering, Zhejiang University Hangzhou 310027 China pengxinheng@zju.edu.cn

## Abstract

A facile strategy to encapsulate R-phycoerythrin (R-PE) proteins and CdSe_*x*_S_1−*x*_/ZnS quantum dots (QDs) in ZIF-8 thin films is developed through a one-pot solid-confinement conversion process. The resultant R-PE/CdSe_*x*_S_1−*x*_/ZnS@ZIF-8 thin film exhibits high-quality white light emission and good thermal stability up to 80 °C.

## Abstract 2

The nanoconfined R-phycoerythrin protein in ZIF-8 shows dual color emissions and exhibits high-quality white light emission and good thermal stability.

## Graphical abstract

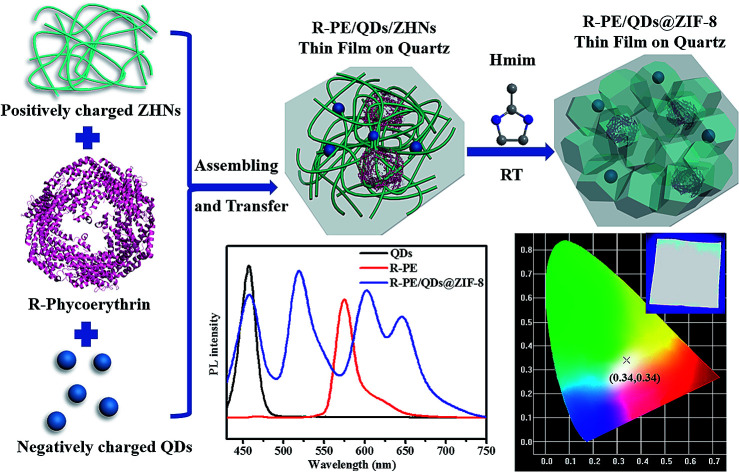

White light-emitting diodes (WLEDs) have been deemed as a promising alternative to incandescent light bulbs as well as fluorescent lamps due to their high efficiency, long lifetime, and environmental friendliness.^1–3^ In general, there are two main strategies to fabricate WLEDs.^4^ One is to combine individual green, red and blue LEDs. Nevertheless, this suffers from high costs, electrochemical corrosion-induced degradation and poor color stability, which restrict their further applications.^5,6^ The other strategy commercially accepted is the integration of a blue- or UV-LED chip with color-conversion phosphors based on an additive color mixing principle. However, the different degradation rates between the individual chips and phosphors results in a relatively low efficiency, chromatic aberration and complicated fabrication processes.^7,8^ Therefore, white light-emitting materials, which can be prepared from a single phase, are thus highly preferred. Furthermore, down-conversion materials, as explained before, must also satisfy the high luminous efficacy of radiation, thermal stability, absorption of the LED wavelength and photo-stability.^9^

Fluorescent proteins (FPs)^10^ with a complicated polypeptide structure^11,12^ have many remarkable luminescence properties, such as a narrow emission line width, good photo-stability and outstanding photon flux saturation.^13^ Ever since the discovery of FPs in 1962,^14^ they have been utilized for live-cell imaging,^15,16^ protein labelling^17^ and environmental biosensors.^18,19^ Lately, these versatile molecules have been applied in lighting devices.^3,20–22^ Notably, FPs have been found to be a promising candidate toward eco-friendly white lighting sources, since the disposal or recycling of FPs causes negligible environmental negative effects. However, their poor thermal stability and requirements for an aqueous environment strongly restrict their applications in WLEDs.^23^ To tackle these issues, Costa and co-workers reported a novel method to achieve FP-based WLEDs with a novel coating system using green, blue and red fluorescent protein-based rubber materials.^3^ Very recently, they demonstrated FP-based WLEDs with a micro-patterned single layer by means of a 3D plotting technique, realizing *x*/*y* color coordinates of 0.33/0.33 and a CCT of 5500 K.^21^ In contrast, Nizamoglu and co-workers directly integrated green and red fluorescent proteins on blue chips which led to a cold white light with a CCT of 8400 K.^22^ Significant achievements have been made in FP-based WLEDs to obtain high-quality white light, however two or three colors of fluorescent proteins are frequently used following a complicated preparation process.

R-phycoerythrin (R-PE) is a kind of fluorescent protein, which carries two types of chromophores including phycoerythrobilin (PEB) and phycourobilin (PUB).^24–27^ Generally, the UV-vis absorption peak of R-PE at 498 nm is attributed to PUB and those at 540 nm as well as 560 nm are attributed to PEB. In pH 6.8 and 0.1 M phosphate buffer, R-PE emitted strong fluorescence at 578 nm due to the energy transfer between PEB and PUB.^28,29^ If the energy transfer between PEB and PUB is impeded,^26^ it is possible to produce dual color emissions from R-PE proteins.

Not only the fluorescent proteins but also the matrix of the FP composite membranes determine the resulting optical performance and thermal stability. Therefore, a matrix with high thermal stability and good transparency, and one that can have FPs easily and uniformly introduced into it is critical for FP-based WLEDs. Metal–organic frameworks (MOFs),^30,31^ which are self-assembled from organic ligands and metal ions, have become highly promising organic–inorganic hybrid materials due to their permanent porosity, tuneable structures and diverse properties.^32–34^ The porous structures of MOFs make them an ideal support for protein encapsulation. Among the thousands of kinds of MOF, ZIF-8 with well-defined cavities (11.6 Å) and a hydrophobic surface stands out due to its excellent thermal stability (up to 823 K), good hydrothermal and chemical stability, and high transparency within the visible range,^35–38^ which make it a promising support for R-PE to fabricate WLEDs. However, MOF crystals applied in lighting devices are usually in the form of powders, which require an extra complicated process to coat them on a blue- or UV-LED chip.^5,39,40^

Herein, following a similar strategy, we synthesized film-like WLEDs by encapsulating R-PE proteins and blue-emitting (B) semiconductor QDs into a ZIF-8 thin film (R-PE/QDs@ZIF-8) at room temperature through a very simple one-pot solid-confinement conversion process.^41,42^ The nanofibrous zinc hydroxide nanostrands (ZHNs) not only serve as a zinc source but also firmly confine R-PE and the blue QDs during ZIF-8 growth by reacting with methyl-imidazole (Hmim). As a result, R-PE confined in ZIF-8 dominantly emits green (518 nm) and red light (650 nm). The single orange light (578 nm) of the original R-PE was dramatically suppressed. This is probably because the polypeptide structures of R-PE are pulled apart due to electrostatic-assisted assembly along the ZHNs, and the formation of PEB–Zn and PUB–Zn complexes.^26,27,29,43–45^ The resultant R-PE/QDs@ZIF-8 thin film exhibits a high photoluminescence quantum yield (PLQY) of 29.8% as R-PE and the QDs are well isolated in the ZIF-8 thin film, which restricts aggregation-caused PL quenching.^4,5^ What’s more, the prepared thin film was processed into a high-quality warm-white LED with ideal CIE coordinates of (0.34, 0.34), a high CRI value of 85 and a moderate CCT value of 4955 K. This process provides a new method to manipulate the fluorescence colors and enhance the thermal stability of FP based-thin films for WLEDs.

The R-PE@ZIF-8 thin film was synthesized through a solid confinement conversion process by *in situ* encapsulation of R-PE into the ZIF-8 thin film at room temperature (Scheme 1). The zinc hydroxide nanostrands (ZHNs) were synthesized according to our previous procedure.^4,41,42^ Then, a different volume of R-PE aqueous dispersion (24.4 μg mL^−1^) was added into 10 mL ZHN solution. The highly positively charged ZHNs^46^ composed of hexagonal clusters of [Zn_61_(OH)_116_(H_2_O)_*n*_]^6+^ could attract R-PE by electrostatic interaction and assemble into the R-PE/ZHNs composite nanofiber dispersions. During this process, R-PE proteins were assembled along the ZHNs, which was similar to the mixture of cadmium hydroxide nanostrands and horse spleen ferritin.^47^ After filtering the R-PE/ZHNs composite nanofiber dispersion onto a polycarbonate (PC) membrane with a pore size of 200 nm, R-PE was evenly embedded in the synthesised R-PE/ZHNs composite thin film. Afterwards, this R-PE/ZHNs composite thin film was transferred onto a quartz plate by being carefully peeled off in ethanol, and immersed into a 25 mM methyl-imidazole (Hmim) ethanol/water solution (volume ratio 1 : 4) for 24 hours. During the ZIF-8 growth process, the top ZHN layer was first converted into a ZIF-8 layer. With an increase in time, the ZIF-8 layer became dense and continuous, which prohibited the release of R-PE proteins. Eventually, a very nice R-PE@ZIF-8 thin film was obtained (Fig. 1) and the R-PE proteins were encapsulated into the ZIF-8 crystal matrix (see details in the Experimental section in the ESI†).

**Scheme 1:**
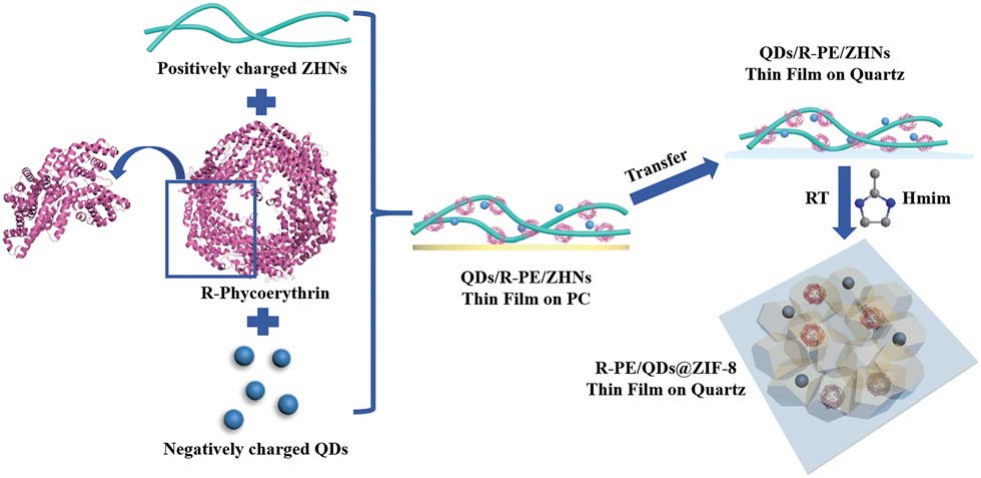
Schematic illustration of the synthesis process of the R-PE/QDs@ZIF-8 thin films on quartz.

**Fig. 1:**
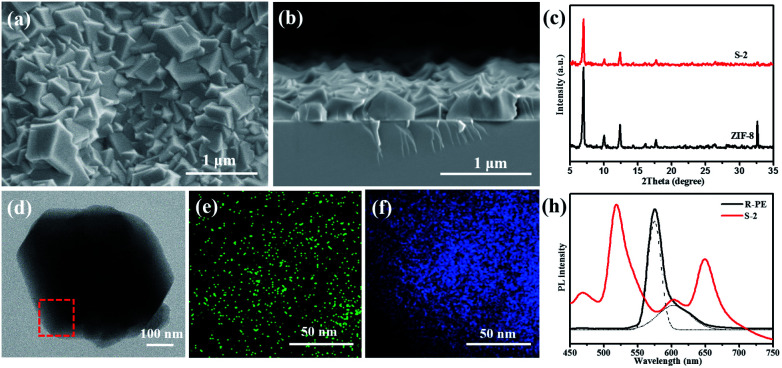
Structure characterization and fluorescent properties. (a) Surface and (b) cross-section SEM images of the R-PE@ZIF-8 thin (S-2) film; (c) XRD patterns of ZIF-8 and the R-PE@ZIF-8 (S-2) thin film; (d) TEM and (e) S element and (f) Zn element mapping of one crystal scraped from the R-PE@ZIF-8 (S-2) thin film; (g) PL spectra of the R-PE@ZIF-8 (S-2) thin film and the same amount of R-PE aqueous solution excited at 405 nm.

In order to investigate the effect of R-PE concentration on the structure and optical properties of the prepared thin films, R-PE@ZIF-8 thin films with different R-PE amounts of 6.5, 10 and 13.5 wt% were prepared and were respectively named S-1, S-2 and S-3 (Table S1†). It is clear that all of the R-PE@ZIF-8 thin films were well-intergrown and continuous with thicknesses of approximately 400 nm (Fig. 1a and b and S1†). Additionally, the representative ZIF-8 crystal phase was formed, as proven by the XRD results (Fig. 1c and S1d†). This indicates that the incorporation of R-PE proteins has no effect on the phase of the ZIF-8 crystals. However, the grain size of the ZIF-8 crystals in the R-PE@ZIF-8 thin film decreased with the increment of the incorporated R-PE amount (Fig. S1a–c†). This might mean that the polar functional groups of the R-PE proteins may easily complex with zinc ions and provide a nucleation site for ZIF-8 growth. Therefore, the more protein included, the more nucleation sites provided, resulting in a smaller size of ZIF-8 crystal.

The TEM element mapping images (Fig. 1d–f) show that the sulfur element from the thio-ether bonds of the R-PE proteins is evenly distributed in the ZIF-8 crystals.^29^ This indicates that the R-PE proteins are uniformly encapsulated and well distributed in the ZIF-8 thin film. As expected, when the R-PE content in the ZIF-8 thin films increased from 6.5 wt% (S-1) to 10 wt% (S-2), the PL intensity of the prepared films rose accordingly (Fig. S1e†). However, when the content of R-PE further increased to 13.5 wt% (S-3), the PL intensity did not increase but was slightly weaker than that of S-2 R-PE@ZIF-8 with an R-PE content of 10 wt%. This means that if the content of R-PE is too high in the ZIF-8 matrix, this will cause aggregation-induced PL quenching.^4,5^ Furthermore, the PL intensities of all of the R-PE@ZIF-8 thin films were far stronger than that of a drop-casted R-PE thin film on quartz with the same amount (0.0927 mg) of R-PE encapsulated in S-2 with the same diameter due to the aggregation-caused PL quenching in the R-PE casted film.^4,5^ In addition, ZIF-8 itself had no obvious fluorescence emission in the emitting range of the R-PE proteins (Fig. S1e†).

It is interesting that, after confining the R-PE proteins in the ZIF-8 matrix, two new fluorescence emission peaks at 518 nm (green) and 650 nm (red) in the visible region appeared, and the emitting peak at 578 nm (orange) was significantly suppressed. Two other weaker peaks at 468 nm and 603 nm are in the alignment with that of the pure R-PE dilute solution (Fig. 1g). The UV-vis absorption spectra of the R-PE@ZIF-8 thin film (S-2) and the pure R-PE solution (Fig. S1f†) clearly present a significant red-shift of the absorption peaks at 498 nm to 509 nm and 565 nm to 587 nm, respectively. The reason for this phenomenon is probably the interaction between the Zn ions and the chromophores of the R-PE proteins. It has been proven that after the formation of complexes PEB–Zn and PUB–Zn, with a tetrahedral structure of which the Zn ion is located at the center, the energy of the π* orbital decreased, and the π → π* and n → π* absorptions were redshifted.^26,27,29,43–45^ The absorption wavelength maximum of PUB was shifted to 509 nm, which is consistent with that of PUB–Zn salts reported in the literature (509 nm). The absorption wavelength maximum of PEB shifted to 597 nm and is also close to that of PEB–Zn salts reported in the literature (583 nm),^43^ indicating the formation of complexes PEB–Zn and PUB–Zn. The absorption peak of PUB–Zn is due to the green fluorescence emission (518 nm) from the R-PE@ZIF-8 thin films. It has been reported that the vanish of around 510 nm emission but the enhancement of 578 nm emission of the R-PE solution is due to the energy transfer from PUB to PEB.^28,29,47^ Therefore, the maximum emission peak of the R-PE@ZIF-8 thin films at 518 nm was probably because ZIF-8 pulls the polypeptide structure of the R-PE molecules apart, and the energy transfer between PEB and PUB was thus blocked.^26^ Furthermore, the rigid structure of the R-PE proteins became loose during this process, so that it was much easier for the Zn ions to get into the proteins and react with the chromophores including PEB and PUB. The absorption peak of PEB–Zn resulted in the emission peak of the R-PE@ZIF-8 thin films at 650 nm through a red-shift.^27,43^ Besides, different local nano-environments could affect the fluorescence emission characteristics of the R-PE molecules. This might be another reason for the red-shift.^48^ Remarkably, the PLQY of R-PE@ZIF-8 (S-2) with 10 wt% R-PE was close to 20%, which was higher than the 11.8% of the same amount of R-PE aqueous dispersion (Table S2†), on account of the well-isolated distribution of the R-PE molecules in the ZIF-8 matrix and the blocking of energy transfer between PEB and PUB. Now, the only orange light-emitting R-PE can emit nice green and red light simultaneously with a high PLQY, which is desirable for building red, green and blue based WLEDs by combining a blue light source.^7,8^

To investigate whether the metal in the MOFs could affect the emission properties of the composite films, R-PE@HKUST-1 membranes with different R-PE amounts of 3, 7 and 12 wt% were prepared and respectively named S-4, S-5 and S-6 (see details in the Experimental section in the ESI†). The surface SEM images of S-4 and S-6 (Fig. S2a and b†) show that all of the membranes are compact and continuous. The cross-sectional SEM image of S-4 (Fig. S2c†) shows that the R-PE@HKUST-1 thin film was well-intergrown with a thickness of approximately 4 μm. In addition, the XRD patterns of the S-4 and S-6 (Fig. S2d†) thin films were in accordance with those of pristine HKUST-1, implying that the incorporation of R-PE does not affect the crystalline phase of HKUST-1. Similar to R-PE@ZIF-8, with the increment of the R-PE content in the composite films, the PL intensities of R-PE@HKUST-1 increased first and then decreased (Fig. S3a†). This might be because if the R-PE content is too high in the HKUST-1 matrix, this will cause aggregation-induced PL quenching.^4,5^ Interestingly, after encapsulating R-PE into the HKUST-1 crystals, a new fluorescence emission peak at 515 nm in the visible region appeared and the emission peak of R-PE at 578 nm was significantly suppressed, which is different from R-PE@ZIF-8 (Fig. S3b†). Therefore, the metal in the MOFs could affect the emission properties of the composite thin films. To obtain the FP-WLEDs, we chose R-PE@ZIF-8 which could emit dual color fluorescence and ZIF-8 has relatively high transparency in the visible range.

Before applying the R-PE@ZIF-8 thin films in the WLED field, their thermal stability should be taken into consideration.^1,4,6,11^ As mentioned before, ZIF-8 with a hydrophobic surface exhibits excellent thermal and hydrothermal stability, and is a suitable host for FP-based WLEDs.^35–38^ Therefore, the optical thermal stability of R-PE encapsulated in ZIF-8 is important. As is well known, fluorescent proteins are not stable at high temperatures.^49,50^ This was proven when we heated the R-PE dilute solution at 80 °C for different durations. As shown in Fig. S4d,† the fluorescence of R-PE at 578 nm in aqueous buffer solution degraded quickly with an increase in duration. After 1 hour, it had already degraded basically, and the characteristic peaks completely disappeared. Nevertheless, after encapsulating the R-PE molecules into ZIF-8, Fig. S4c† shows that the degradation of the PL intensity of R-PE@ZIF-8 (S-2) at 518 nm is only 0.92% after treatment at 80 °C for 3 hours in air, and 6.5% for 10 hours. This means that the encapsulation of the R-PE proteins into ZIF-8 could significantly enhance their optical stability. In addition, after thermal treatment, the crystal structure of R-PE@ZIF-8 (S-2) was reserved (Fig. S4a and b†). It has been mentioned that the poor thermostability of FPs limits their wide applications in the lighting field. Fortunately, this problem was resolved when we encapsulated the R-PE molecules into the ZIF-8 crystals.

Although the fluorescence emission of the R-PE@ZIF-8 thin films included nice green and red light, they could not emit white light on account of lacking blue light. With this in mind, blue CdSe_*x*_S_1−*x*_/ZnS QDs with fluorescence emission at 458 nm excited under 405 nm were added into the R-PE@ZIF-8 thin films through a similar process (Scheme 1 and more details are in the Experiment section in the ESI†). Typically, the highly positive ZHN solution (10 mL) was mixed with a certain volume of R-PE aqueous dispersion (0.0244 mg mL^−1^) and negatively charged QD (0.1 mg mL^−1^) dilute solution. The R-PE molecules and QDs subsequently assembled onto the surface of the ZHNs to form R-PE/QDs/ZHNs composite nanofibers by electrostatic interactions. Then an R-PE/QDs/ZHNs composite film was formed by filtering the R-PE/QDs/ZHNs composite nanofibers on a PC membrane with a pore size of 200 nm. After transferring the composite nanofibrous thin film from the PC to a quartz substrate, it reacted with imidazole to form an R-PE/QDs@ZIF-8 thin film on quartz.

To obtain white light-emitting thin films, we prepared R-PE/QDs@ZIF-8 thin films with different CdSe_*x*_S_1−*x*_/ZnS QD amounts of 3, 5, 10 and 13 wt% but kept the R-PE content as 10 wt%, and these were respectively named S-7, S-8, S-9 and S-10. It was obvious that the R-PE/QDs@ZIF-8 thin film (S-9) was continuous and well-intergrown (Fig. 2a and S5†). Furthermore, the cross-section element mapping images show that the blue QDs are distributed homogeneously in the thin film with a thickness of 500 nm (Fig. 2b). In addition, the TEM images (Fig. 2c and h) and element mapping (Fig. 2d–g) further confirmed that the QDs were incorporated into the ZIF-8 crystals. The high-resolution TEM (HRTEM) image (Fig. 2i) indicated that the QDs with a diameter of approximately 4 nm were evenly encapsulated in the ZIF-8 crystals. Not surprisingly, a ZIF-8 crystal phase was obtained, as confirmed by the XRD results (Fig. S6†).

**Fig. 2:**
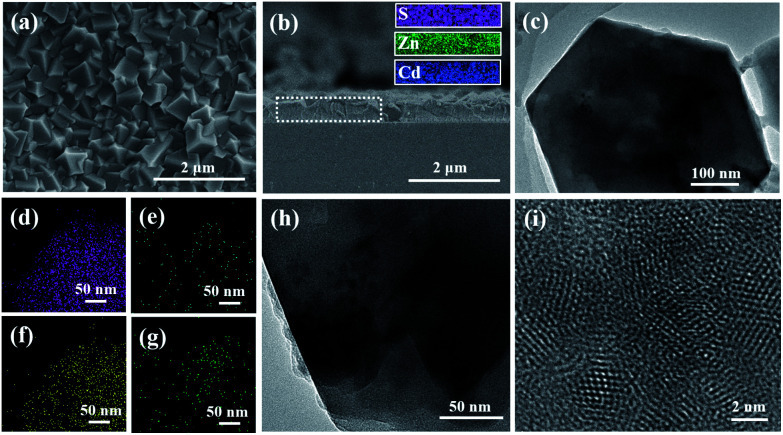
Characterization of the R-PE/QDs@ZIF-8 thin film (S-9). (a) Surface and (b) cross-section SEM images; (c) TEM image and the corresponding element mapping for (d) Zn, (e) Cd, (f) N and (g) S, respectively; (h) high magnification and (i) HRTEM images. The insets in (b) are the corresponding element mapping of S, Zn, and Cd.

Obviously, upon excitation at 405 nm, the R-PE/QDs@ZIF-8 thin film (S-9) emitted blue, green, and red light at 450 nm, 518 nm, 603 nm and 650 nm, respectively (Fig. 3a). The reasons for the appearance of green and red fluorescence emission peaks have been explained before. In addition, the characteristic emission at 458 nm was consistent with the PL emission of the blue QD dilute solution. Notably, the addition of QDs further enhanced the fluorescence emission of R-PE through some uncertain energy transfer, and this function would be strengthened by increasing the QD content (Fig. 3b). Compared to the same amount of the R-PE/QDs dilute solution, the R-PE/QDs@ZIF-8 thin film exhibited a higher PLQY up to 29.8% (Table S2†), indicating that the QDs and R-PE molecules were well isolated and evenly encapsulated in the ZIF-8 thin films to decrease the aggregation-caused PL quenching.

**Fig. 3:**
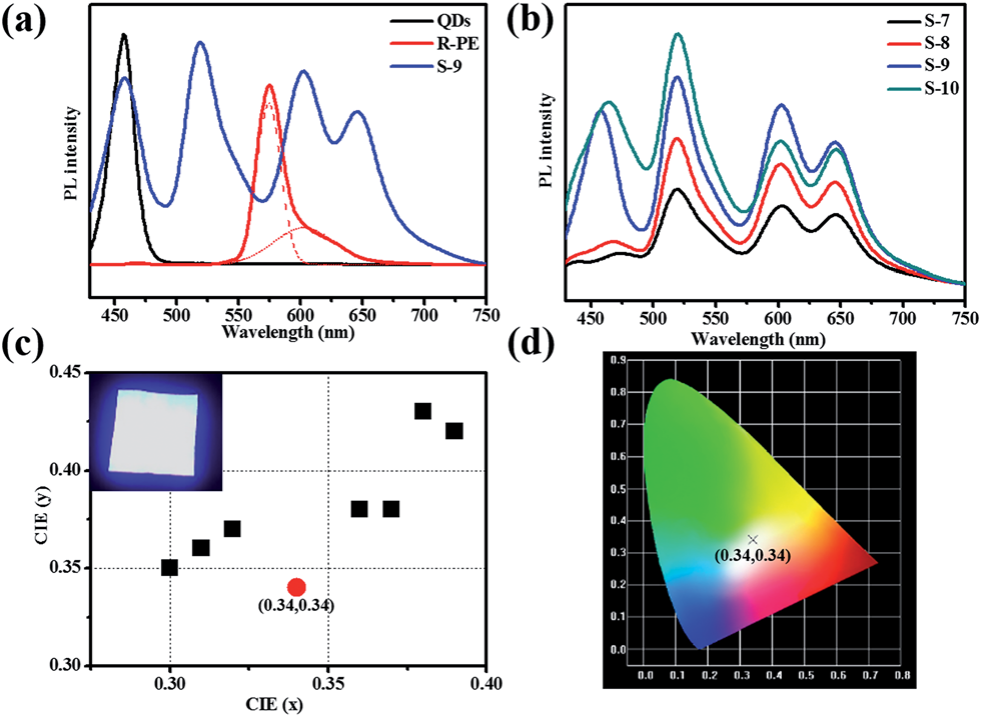
Optical properties of the R-PE/QDs@ZIF-8 thin films. (a) PL spectra of the R-PE/QDs@ZIF-8 thin film (S-9), the R-PE solution, and the QD solution with the same amount excited at 405 nm. (b) PL spectra of the R-PE/QDs@ZIF-8 thin films (S-7 to S-10) with different amounts of R-PE and QDs excited at 405 nm. (c) CIE coordinates of the R-PE/QDs@ZIF-8 thin films (S-7 to S-14) with different amounts of R-PE and QDs excited at 405 nm. The inset photo in (c) is a photograph of the white light-emitting LEDs assembled from the R-PE/QDs@ZIF-8 thin film (S-9) with UV-violet (405 nm) LED curing chips at the on state. (d) Emission colors in the CIE 1931 chromaticity diagram of the R-PE/QDs@ZIF-8 thin film (S-9) excited at 405 nm.

After carefully optimizing the concentration of the QDs, a R-PE/QDs@ZIF-8 thin film with an R-PE content of 10 wt% and a blue QD content of 10 wt% was prepared and named S-9. Under 405 nm excitation, the fluorescence emission of this thin film could cover the whole visible spectrum, leading to white light emission (Fig. 3b and d). For application as a WLED, we placed R-PE/QDs@ZIF-8 (S-9) prepared on quartz directly upon a UV-violet (405 nm) LED chip array. As a result, the Commission Internationale de I’E’clairage (CIE) coordinates of the R-PE/QDs@ZIF-8 thin film were (0.34, 0.34), which are very close to the ideal white-light emission (0.33, 0.33). By putting the resultant film into an integrating sphere, the absolute quantum yield (QY) of S-6 was measured as 29.8%, which is much higher than the 11.1% of the R-PE/QDs dilute solution with the same amount (Table S2†). Besides, the correlated color temperature (CCT) was 4955 K, and the color rendering index (CRI) was approximately 85. Compared to the solar spectrum at 5000 K (Fig. S7†), the fluorescence emission spectrum of the R-PE/QDs@ZIF-8 thin film (S-9) was basically consistent with sunlight, which is suitable for human eyes. Based on these results, we concluded that the R-PE/QDs@ZIF-8 thin film could emit high-quality white light, and might find a potential application in practical lighting devices.

In conclusion, we have demonstrated a one-pot solid-confinement conversion process to encapsulate R-PE molecules and blue quantum dots into ZIF-8 crystals for the design of high-quality white-emitting thin films. The R-PE molecules and QDs are well isolated and evenly distributed in the ZIF-8 crystals, leading to a higher PLQY by suppressing aggregation-caused PL quenching. Interestingly, the chromophores of R-PE could form complexes with Zn ions. Meanwhile, the crystal growth process might block the energy transfer between these two types of chromophore, so that R-PE could emit green and red colors in the visible region, replacing the single orange color from its solution. Furthermore, the thermal stability of R-PE embedded in the ZIF-8 films at high temperatures has been improved significantly. Upon excited at 405 nm, the resultant R-PE/QDs@ZIF-8 thin film emits high-quality warm white light with CIE coordinates of (0.34, 0.34), a CRI of 85, and a CCT of approximately 4955 K, thus demonstrating its promising applicability for color-conversion based warm W-LEDs. This solid-confinement conversion process could also be used to encapsulate other fluorescent proteins or phosphorous molecules in metal–organic frameworks to realize high-quality white-light emission for applications in lighting devices.

## Conflicts of interest

There are no conflicts to declare.

## Supplementary Material

RA-009-C9RA00161A-s001
