# Supplementary material for: Dual emission from nanoconfined R-phycoerythrin fluorescent proteins for white light emission diodes
Source: RSC Adv. 2019 Mar 27;9(17):9777–82. doi: 10.1039/c9ra00161a (PMC9062197; doi:10.1039/c9ra00161a)
Supplement: RA-009-C9RA00161A-s001 [file RA-009-C9RA00161A-s001.pdf]

Electronic Supplementary Material (ESI) for RSC Advances.
This journal is © The Royal Society of Chemistry 2019

SUPPORTING INFORMATION

Duel Emissions from Nanoconfined R-phycoerythrin Fluorescent Proteins for White Light Emission Diodes

Xiaobin Wang, Yi Guo, Zhuoyi Li, Wen Ying, Danke Chen, Zheng Deng and Xinsheng Peng*

Abstract: The requirements for an aqueous environment and low temperature of fluorescent proteins (FPs) have strongly restricted their wide applications in lighting devices. Herein, we present a facile strategy to encapsulate R-Phycoerythrin (R-PE) proteins and $CdSe_xS_{1-x}$/ZnS quantum dots (QDs) in ZIF-8 thin films through a one-pot solid-confinement conversion process. Due to the confinement, the R-PE@ZIF-8 membrane presents dual colors emission at green (518 nm) and red (602, 650 nm), respectively, while the orange emission (578 nm) of pure R-PE is significantly suppressed. By co-encapsulating $CdSe_xS_{1-x}$/ZnS QDs with blue fluorescence emission (458 nm) into the ZIF-8 crystals, the resultant R-PE/$CdSe_xS_{1-x}$/ZnS@ZIF-8 thin film exhibits high-quality white light emission with Commission Internationale de l'E'clairage (CIE) coordinates of (0.34, 0.34), a color-rendering index (CRI) of 85, a correlated color temperature (CCT) of approximately 4955 K and good thermal stability up to 80 ℃.

1. Experiment details:

Materials and chemicals: Zinc nitrate hexahydrate ($Zn(NO_3)_2$•6H2O), 1,3,5-benzenetricarboxylic acid (trimesic acid, H_3BTC) and ethanol were purchased from Sinopharm Chemical Reagent Co. Ltd. Copper nitrate ($Cu(NO_3)_2 \cdot 3H_2O$) and ethanolamine (AE) was purchased from Acros Chemicals. 2-Methylimidazole (Hmim) was purchased from aladdin. The R-Phycoerythrin proteins were purchased from BioVision supplied in 100 mM Phosphate buffer with concentration of 12.5 mg mL^{-1}. The R-PE molecules emit 578 nm orange fluorescence at 405 nm excitation. $CdSe_xS_{1-x}$/ZnS QDs nanocrystals colloids were purchased from Najing Tech as aqueous dispersions of 1 mg mL^{-1}. The nanocrystals have 458 nm blue fluorescence emission under 405 nm excitation.

Preparation of ZHNs: Zinc hydroxide nanostrands (ZHNs) was synthesized by quickly mixing 8 mM $Zn(NO_3)_2$ ethanol/water (volume 2:3) solution with 1.6 mM AE ethanol/water (volume 2:3) solution and then stirred for about 5 minutes as well as left at room temperature for 30 minutes.

Preparation of R-PE@ZIF-8 thin films: To prepare R-PE@ZIF-8 thin films with different content, ZHNs was mixed with different volumes of R-PE dilute solution with concentration of 0.0244 mg mL^{-1}. 10 mL ZHNs was mixed with 2.5, 3.8, or 5 mL stocked R-PE to prepare R-PE@ZIF-8 thin films with R-PE content of 6.5 wt%, 10 wt%, or 13.5 wt%, respectively (Table S1). After filtering the R-PE/ZHNs composite dispersion on PC membrane with the pore size of 200 nm, this composite membrane was further transferred onto a quartz plate by carefully peeling off in ethanol, and immersed into 10 mL of 25 mM methyl-imidazole (Hmim) ethanol/water solution (volume ratio 1:4) for 24 hours at room temperature. The R-PE@ZIF-8 membrane with 10 wt% R-PE was explored to study the thermal stability at 80 ℃ for different time.

Preparation of CHNs: Copper hydroxide nanostrands (CHNs) was synthesized by quickly mixing equal volume 4 mM copper nitrate aqueous solution with 1.6 mM AE aqueous solution and then stirred for about 5 minutes as well as left at room temperature for 24 hours.

Preparation of R-PE@HKUST-1 thin films: In order to prepare R-PE@HKUST-1 thin films with different content, CHNs was mixed with different volumes of R-PE dilute solution with concentration of 0.0244 mg mL^{-1}. 30 mL CHNs was mixed with 2.6, 6.2, or 10.5 mL stocked R-PE to prepare R-PE@ZIF-8 thin films with R-PE content of 3 wt%, 7 wt%, or 12 wt%, respectively. Then the mixed solution was filtered on a porous polycarbonate (PC) substrate with a pore size of 200 nm, to form a R-PE/CHNs composite thin film, which was further peeled off from PC substrate and transferred onto a quartz plate. The R-PE@HKUST-1 composite thin films were obtained by immersing R-PE/CHNs composite thin film into H_3BTC ethanol–water (vol:vol=1:1) solution at room temperature for 1 hour.

Preparation of R-PE/QDs@ZIF-8 thin films: To obtain white-light emitting thin films, we prepared R-PE/QDs@ZIF-8 membranes with different content of blue QDs but keeping R-PE content of 10 wt%. ZHNs 10 mL was mixed with 0.28, 0.45, 0.9, or 1.2 mL stocked blue QDs and 3.8 mL stocked R-PE to prepare R-PE/QDs@ZIF-8 thin films with QDs content of 3 wt%, 5 wt%, 10 wt%, or 13 wt% respectively (Table S1). White-light-emitting R-PE/QDs@ZIF-8 thin film was synthesized from 10 ml ZHNs with 0.9mL 458 nm blue QDs aqueous dispersion and 3.8 mL orange-emitting R-PE dilute solution. Before test, all the prepared membranes were dried in air after rinsing with ethanol twice.

Materials characterization: The morphologies and internal structure were characterized by scanning electronic microscopy (SEM) (Hitachi S-4800) and transmission electron microscopy (TEM) (Tecnai G2 F20 S-TWIN). The phase of the resultant films was characterized by X-ray diffraction (XRD) using an X'Pert PRO (SHIMADZU XRD-6000) instrument with a Cu Kα radiation source. Photoluminescence spectra of R-PE@ZIF-8 thin films, R-PE@HKUST-1 thin films and R-PE/QDs@ZIF-8 membranes were recorded by spectrophotometer (Edinburgh Instruments FLS920) with a 405 nm laser. The ultraviolet/visible absorption spectra of R-PE@ZIF-8 thin film and R-PE solution were conducted on UV/Vis spectrometer (Agilent Cary 5000). The absolute photoluminescence quantum yields of R-PE dilute solution, R-PE/QDs mixture solution, R-PE@ZIF-8 thin films and R-PE/QDs@ZIF-8 membranes were measured by a spectrometer (Hitachi U 4100) that equipped with an integrating sphere.

Thermal stability: PL spectra of the 578 nm R-PE dilute solution treated in air at 80 ℃ for different time (1 h, 3 h and 10 h) were obtained by spectrophotometer (Edinburgh Instruments FLS920) with a 405 nm laser. The PL spectra of a R-PE@ZIF-8 thin film (S-2) on a quartz plate after treated in air at 80 ℃ for 1 h, 3 h and 10 h, respectively, were also recorded.

White light emission demonstration: The resultant R-PE/QDs@ZIF-8 membrane (from 10 mL ZHNs, 3.8 mL 578 nm R-PE dilute solution and 0.9 mL 458 nm blue QDs aqueous dispersion) on a quartz plate was placed on a UV LED chip arrays (405 nm, purchased from HOYA).

2. Additional SEM images，XRD patterns and PL results.

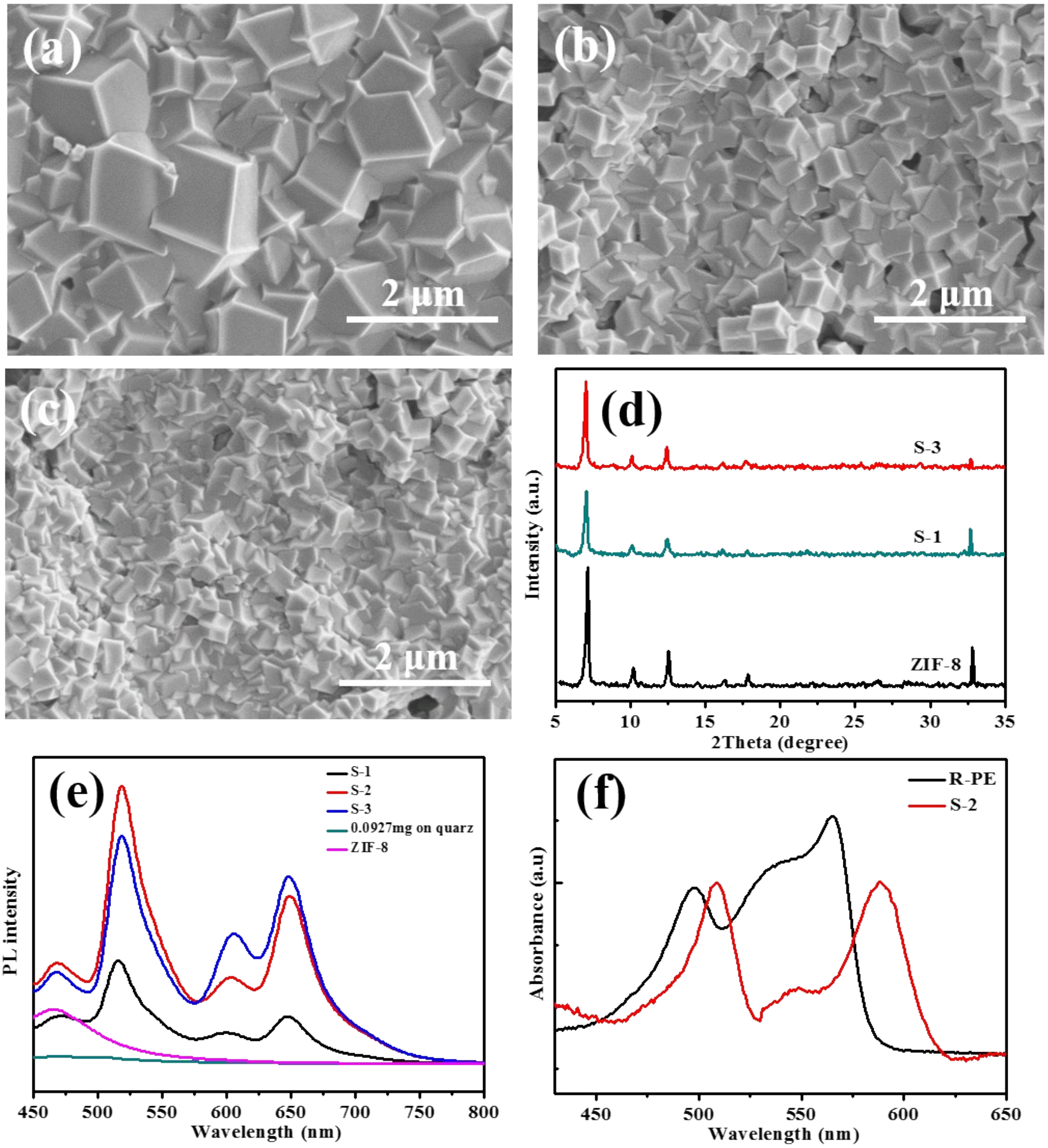

Figure S1. (a),(b) and (c) are the surface SEM images of ZIF-8, S-1 and S-3 R-PE@ZIF-8 thin films, respectively; (d) XRD patterns of ZIF-8, R-PE@ZIF-8 thin films (S-1 and S-3); (e) PL spectra of ZIF-8 thin film, R-PE protein thin film by drop casting on quartz, R-PE@ZIF-8 S-1 to S-3 thin films. (f) The visible range absorption spectra of R-PE solution and R-PE@ZIF-8 thin film (S-2).

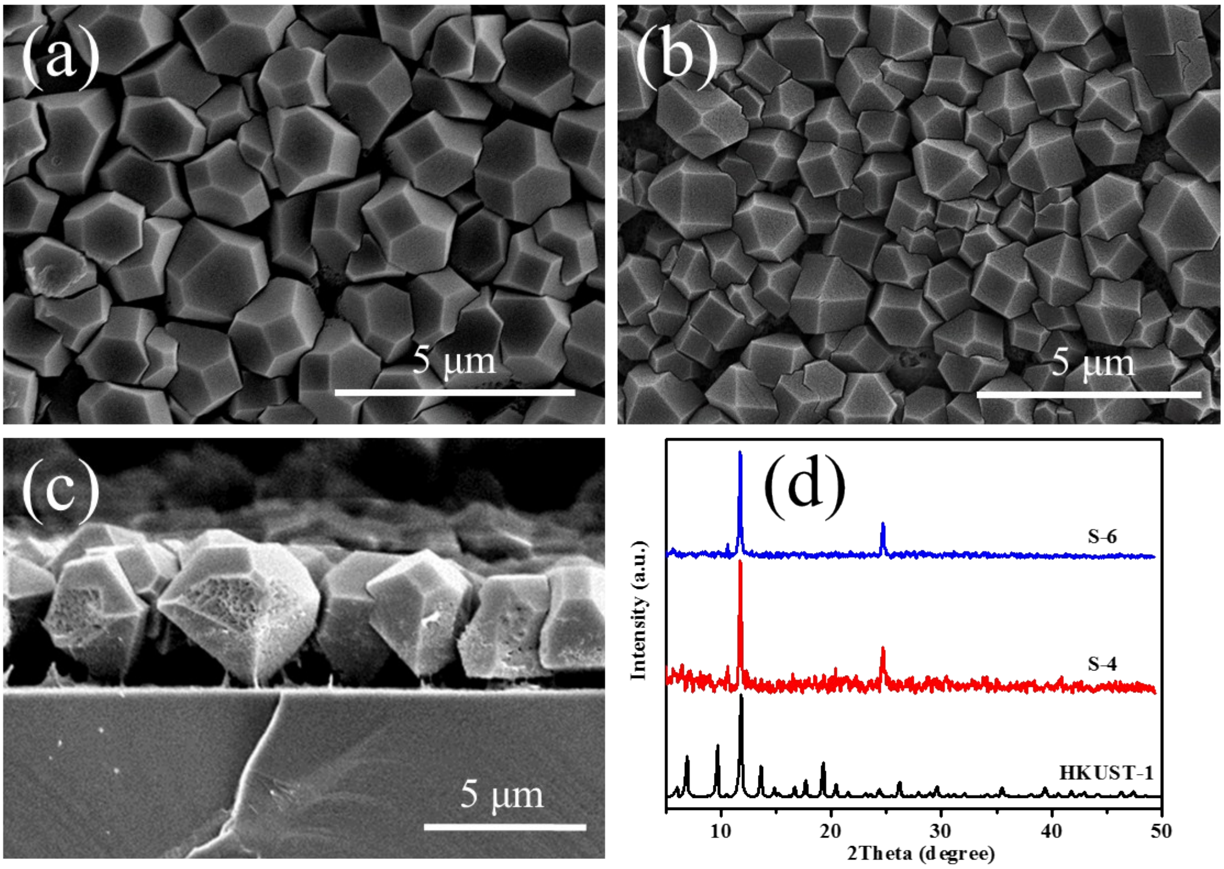

Figure S2. (a), (b) are the surface SEM images of S-4 and S-6 R-PE@HKUST-1 thin films, respectively; (c) is the cross-section SEM image of S-4; (d) XRD patterns of HKUST-1, R-PE@HKUST-1 thin films (S-4 and S-6).

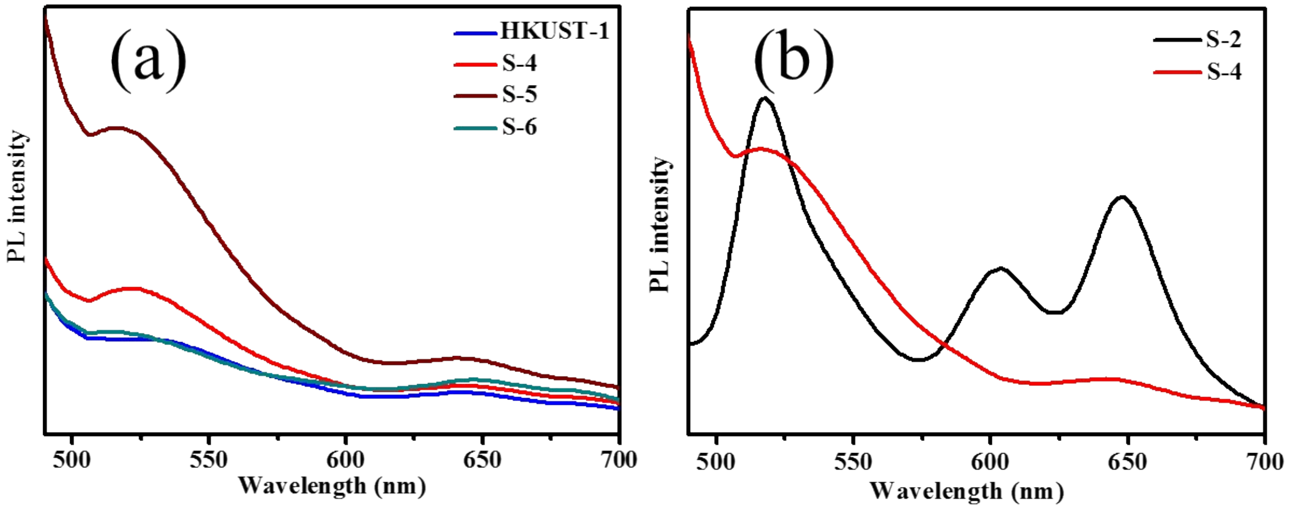

F

igure S3. (a) PL spectra of R-PE@HKUST-1 thin films (S-4 to S-6) with different content of R-PE excited at 405 nm. (b) PL spectra of the R-PE@ZIF-8 thin film (S-2) and the R-PE@HKUST-1 thin film (S-4) excited at 405 nm.

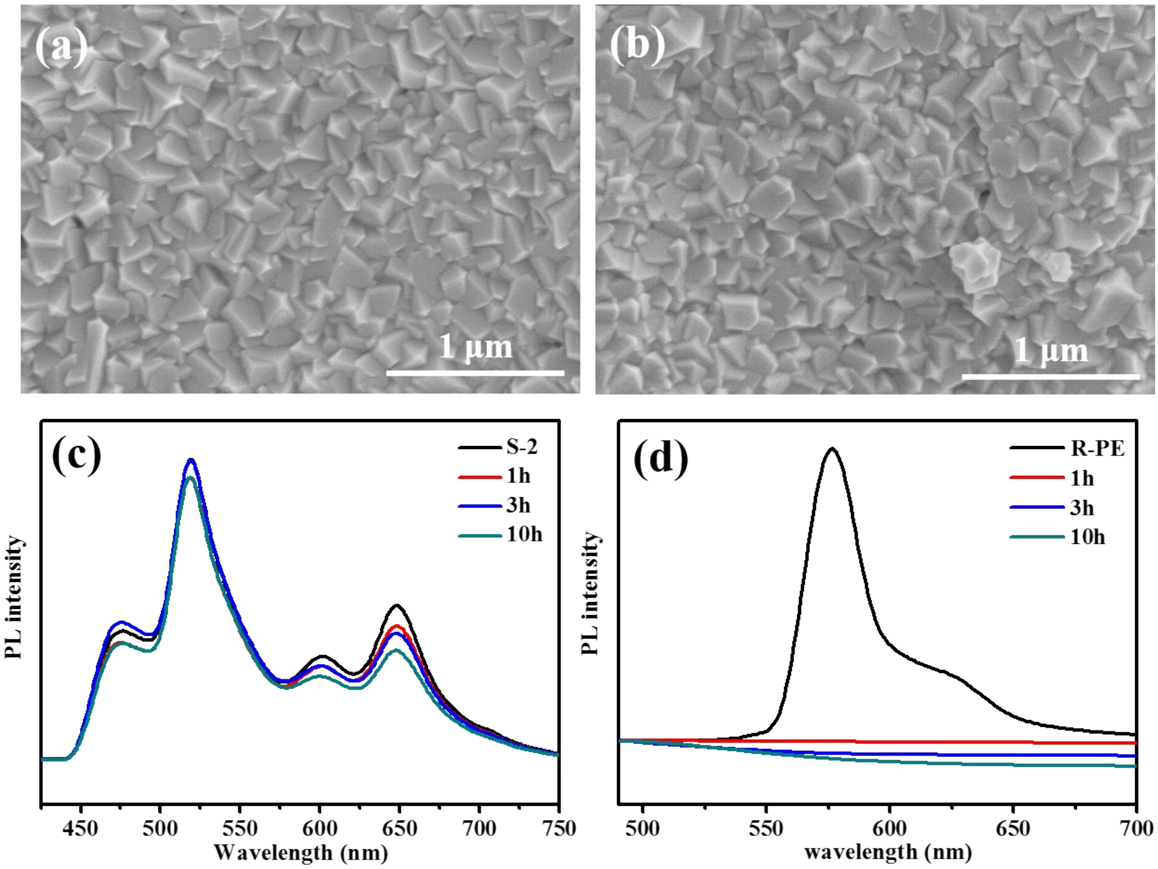

Figure S4. SEM images of (a) original R-PE@ZIF-8 film (S-2) and (b) after treated at 80 °C in air for 10 hours; and the PL stability of (c) R-PE@ZIF-8 thin films (S-2) and (d) R-PE solution with the same amount treated at 80 °C, respectively, excited at 405 nm.

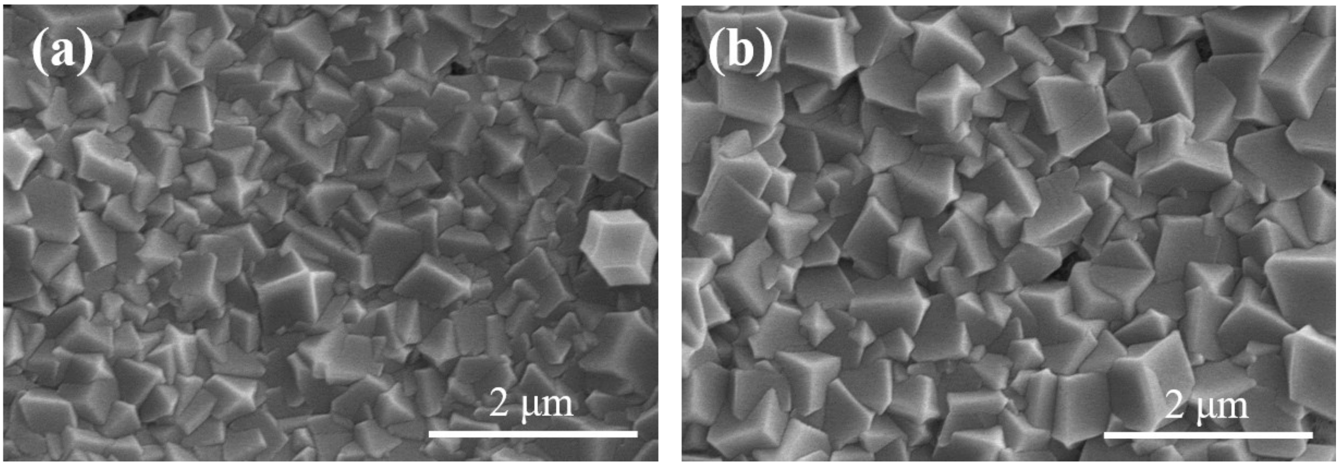

Figure S5. SEM images of R-PE/QDs@ZIF-8 thin films (a) (S-7) and (b) (S-8).

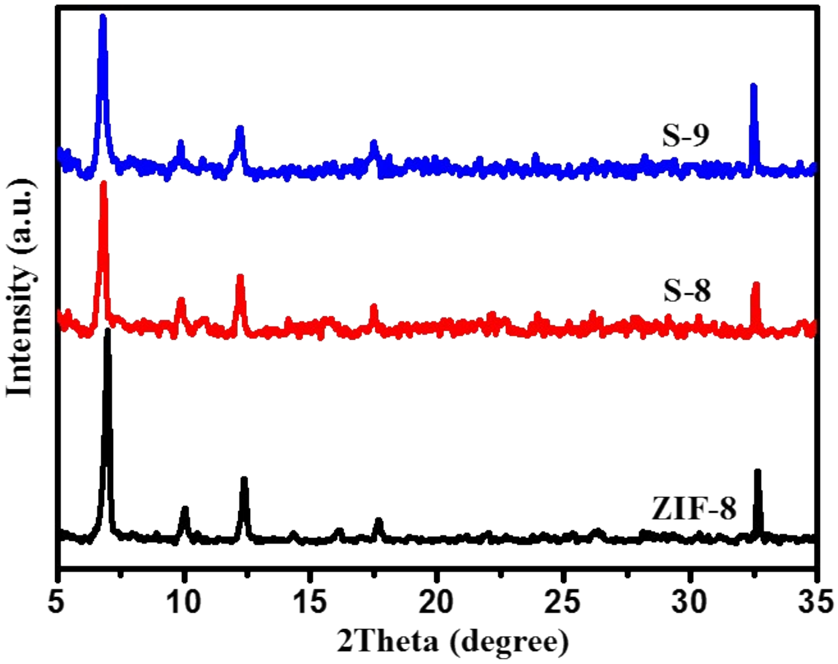

Figure S6. XRD patterns recorded from ZIF-8, R-PE/QDs@ZIF-8 (S-8 and S-9) thin films, respectively.

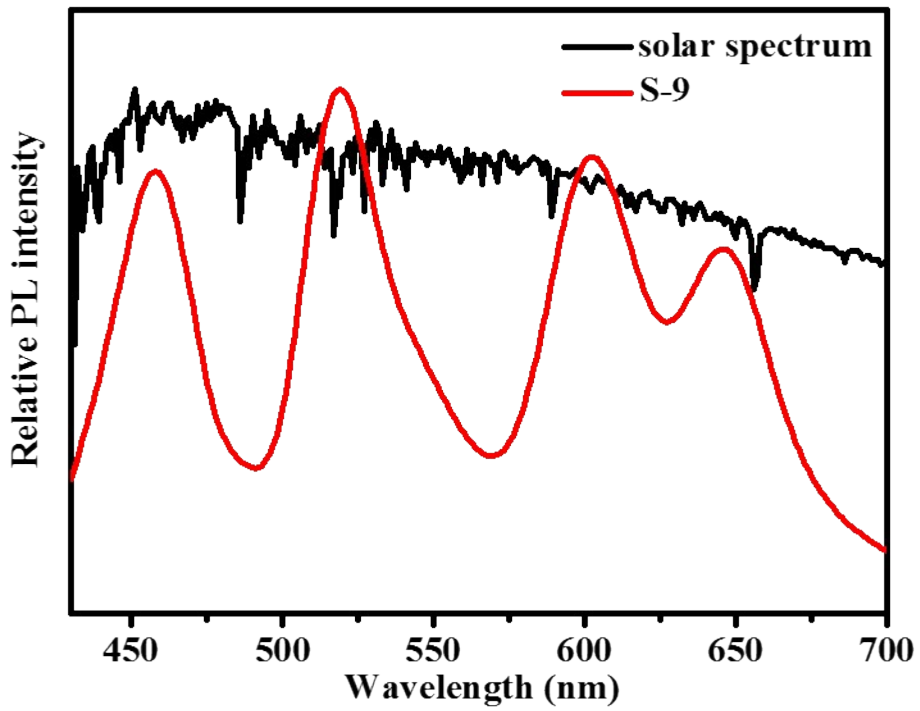

Figure S7. PL spectra of sunlight at 5000 K and the R-PE/QDs@ZIF-8 thin film (S-9).

3. Additional tables:

Table S1. R-PE, QDs content and CIE coodinates of all membranes we prepared.

Sample	R-PE Content wt%	QDs Content wt%	CIE(x)	CIE(y)
1	6.5	-	-	-
2	10	-	-	-
3	13.5	-	-	-
7	10	3	0.38	0.43
8	10	5	0.39	0.42
9	10	10	0.34	0.34
10	10	13	0.30	0.35
11	10	2.5	0.37	0.38
12	10	2	0.36	0.38
13	10	11.5	0.31	0.36
14	10	12	0.32	0.37

Table S2. PL Quantum yield of R-PE solution, R-PE/QDs solution and corresponding membranes.

Sample	Quantum yield
R-PE solution	11.83%
R-PE/QDs solution	11.13%
R-PE@ZIF-8 (S-2)	19.18%
R-PE/QDs@ZIF-8 (S-9)	29.83%
